# Brain-derived neurotrophic factor reduces inflammation and hippocampal apoptosis in experimental *Streptococcus pneumoniae* meningitis

**DOI:** 10.1186/s12974-017-0930-6

**Published:** 2017-08-04

**Authors:** Danfeng Xu, Di Lian, Jing Wu, Ying Liu, Mingjie Zhu, Jiaming Sun, Dake He, Ling Li

**Affiliations:** 1 Department of Pediatric Neurology, Xinhua Hospital affiliated to Shanghai Jiaotong University School of Medicine, Kongjiang Rd 1665, Shanghai, 200092 People’s Republic of China; 2 Department of Clinical Laboratory, Xinhua Hospital affiliated to Shanghai Jiaotong University School of Medicine, Shanghai, 200092 People’s Republic of China; 3 Department of Pathology, Xinhua Hospital affiliated to Shanghai Jiaotong University School of Medicine, Shanghai, 200092 People’s Republic of China

**Keywords:** *Streptococcus pneumoniae* meningitis, Brain-derived neurotrophic factor, Neuroinflammation, Hippocampal apoptosis

## Abstract

**Background:**

*Streptococcus pneumoniae* meningitis is a serious inflammatory disease of the central nervous system (CNS) and is associated with high morbidity and mortality rates. The inflammatory processes initiated by recognition of bacterial components contribute to apoptosis in the hippocampal dentate gyrus. Brain-derived neurotrophic factor (BDNF) has long been recommended for the treatment of CNS diseases due to its powerful neuro-survival properties, as well as its recently reported anti-inflammatory and anti-apoptotic effects in vitro and in vivo.

**Methods:**

In this study, we investigated the effects of BDNF-related signaling on the inflammatory response and hippocampal apoptosis in experimental models of pneumococcal meningitis. Pretreatment with exogenous BDNF or the tropomyosin-receptor kinase B (TrkB) inhibitor k252a was performed to assess the activation or inhibition of the BDNF/TrkB-signaling axis prior to intracisternal infection with live *S. pneumoniae*. At 24 h post-infection, rats were assessed for clinical severity and sacrificed to harvest the brains. Paraffin-embedded brain sections underwent hematoxylin and eosin staining to evaluate pathological severity, and cytokine and chemokine levels in the hippocampus and cortex were evaluated by enzyme-linked immunosorbent assay. Additionally, apoptotic neurons were detected in the hippocampal dentate gyrus by terminal deoxynucleotidyl transferase dUTP-nick-end labeling, key molecules associated with the related signaling pathway were analyzed by real-time polymerase chain reaction and western blot, and the DNA-binding activity of nuclear factor kappa B (NF-κB) was measured by electrophoretic mobility shift assay.

**Results:**

Rats administered BDNF exhibited reduced clinical impairment, pathological severity, and hippocampal apoptosis. Furthermore, BDNF pretreatment suppressed the expression of inflammatory factors, including tumor necrosis factor α, interleukin (IL)-1β, and IL-6, and increased the expression of the anti-inflammatory factor IL-10. Moreover, BDNF pretreatment increased TrkB expression, activated downstream phosphatidylinositol 3-kinase (PI3K)/protein kinase B (AKT) signaling, and inhibited the myeloid differentiation primary response gene 88 (MyD88)/NF-κB-signaling pathway.

**Conclusions:**

These data suggested that BDNF administration exerted anti-inflammatory and anti-apoptotic effects on an experimental pneumococcal meningitis model via modulation of MyD88/NF-κB- and PI3K/AKT-signaling pathways. Our results indicated that treatment with exogenous BDNF might constitute a potential therapeutic strategy for the treatment of bacterial meningitis.

## Background

Bacterial meningitis is a severe infection of the central nervous systems (CNS), with an annual occurrence of 0.9 per 100,000 people in developing countries [1, 2]. The most common causative agent is *Streptococcus pneumoniae*, with a case fatality rate of ~30% in developed countries and nearly 50% in less-developed countries [3–5]. Moreover, ~50% of survivors suffer from persistent neurological sequelae throughout their life, including learning and memory deficits, seizures, and hearing impairment [6, 7]. The inflammatory response plays a vital role in disease pathogenesis, with bacterial compounds recognized by brain-resident immune cells capable of recruiting myeloid differentiation factor 88 (MyD88) and inducing nuclear translocation of nuclear factor kappa B (NF-κB), followed by the production of inflammatory mediators [8, 9]. This enhanced inflammatory response triggered to eliminate bacterial components exerts both defensive and neurotoxic effects [10]. Although therapies necessary to reduce inflammation are required, neurological sequelae associated with the disease correlate with hippocampal apoptosis caused by both the bacterial toxins and the intensive immune response [10]. A single treatment capable of reducing both inflammation and hippocampal apoptosis could potentially improve outcomes in children with *S. pneumoniae* meningitis.

Brain-derived neurotrophic factor (BDNF) is a member of the neurotrophic family, which plays an important role in the development, differentiation, and survival of neurons in the CNS [11, 12]. BDNF exerts neuroprotective effects in multiple CNS diseases following its high-affinity binding to tropomyosin-receptor kinase B (TrkB) [13, 14]. In recent years, significant effort has been expended to identify the neuroprotective effects of BDNF on *S. pneumoniae* meningitis in both animal experiments and clinical studies. Our previous study reported the levels of BDNF and its receptor TrkB increased following acute *S. pneumoniae* meningitis but subsequently declined over time, especially following administration of antibiotics [15]. Similarly, increased BDNF levels were also observed in the serum and cerebrospinal fluid (CSF) of pediatric patients with CNS infections on the day of admission [16]. Increased BDNF synthesis during the acute phase of meningitis could stimulate proliferation of dentate granule cells and promote neurogenesis after bacterial meningitis [17]; however, this self-reparative capacity is insufficient, given that most newly generated cells are unable to differentiate into immature neurons and neurons in experimental *S. pneumoniae* meningitis [9], which worsens as BDNF decreases over time. Additionally, Barichello et al. [18] reported that decreases in BDNF levels during the long-term phase of meningitis were correlated with behavioral deficits in adult animals submitted to meningitis during the neonatal period. Interestingly, our previous study reported that administration of exogenous BDNF increased rates of neuron survival [18], and it was recently reported that exogenous BDNF increases neurogenesis of neuron stem cells in the hippocampus after *S. pneumoniae* meningitis [9]. In addition to its neuroprotective effects, BDNF participates in anti-inflammatory and anti-apoptotic processes according to a study of experimental allergic encephalomyelitis [13]. Furthermore, BDNF can attenuate ischemic-hypoxic injury by modulating local inflammation in rats suffering from ischemic stroke [19]. Taken together, these findings indicate BDNF involvement in regulating inflammatory processes; however, the mechanisms associated with BDNF signaling related to these responses remain unknown. BDNF-related neuroprotective effects are elicited by activation of extracellular signal-related kinase (ERK)- and phosphatidylinositol 3-kinase (PI3K)/protein kinase B (AKT)-signaling pathways, and recent evidence suggests that PI3K participates in negative regulation of inflammatory pathways [13, 20, 21]. However, the contribution of this signaling pathway to BDNF-associated prevention of brain injury related to *S. pneumoniae* meningitis remains unclear.

Here, we explored whether BDNF/TrkB interaction modulates localized inflammation in the infected brain by exerting neuroprotective effects through reductions in hippocampal apoptosis associated with *S. pneumoniae* meningitis. We further investigated whether these effects are mediated by the MyD88/NF-κB- and PI3K/AKT-signaling pathways.

## Methods

### Animals and cannula implantation

Three-week-old female Sprague-Dawley rats (50–55 g) were obtained from the Shanghai Laboratory Animal Management Center (Shanghai, China). Cannula implantation was undertaken based on previously described procedures [9]. Briefly, one stainless steel cannula was implanted into the right lateral cerebral ventricle after the rat was anesthetized with 10% chloral hydrate (0.15–0.3 mL per 100 g, administered intraperitoneally). The location of the cannula implantation was 3.8 mm rostral to the lambdoid suture of the skull, 2 mm lateral to the right side from the midline of the skull, and 2.5 mm from the skull surface. Following surgery, all rats were returned to their cages to allow a 3-day recovery. Rats were housed under a 12-h light/dark cycle, with food and water available ad libitum. Animal experiments were approved by the Animal Ethical and Welfare Committee of Xinhua Hospital affiliated to Shanghai Jiaotong University School of Medicine. All efforts were made to minimize the number of animals used and their suffering.

### Infecting organisms

The standard strain of serotype III *S. pneumoniae* was obtained from American Type Culture Collection (Manassas, VA, USA). The bacterial strain was cultured overnight on a blood agar plate, followed by inoculation into Vital Aer Broth (R&D Systems, Minneapolis, MN, USA) and incubation at 37 °C under 5% CO_2_ for 18 h to reach the logarithmic phase as reported previously [22]. Bacteria were centrifuged for 15 min at 5000*g* and re-suspended in sterile saline solution to an approximate concentration of 1 × 10^4^ CFU/mL by using a nephelometer (bioMerieux, Marcy-l’Étoile, France).

### Animal model of *S. pneumoniae* meningitis

Bacterial inoculations were performed under anesthesia. The rats underwent an intracisternal puncture to remove 20 μL of CSF, followed by intracisternal injection of a 20-μL volume containing either 1 × 10^4^ CFU/mL *S. pneumoniae* or pyrogen-free saline. At 24 h post-inoculation, all infected rats developed meningitis according to clinical evaluation, which was confirmed by quantitative analysis of culturing 5 μL of CSF obtained through a puncture of the cisterna magna. The rats were weighed, and the severity of the disease was assessed clinically using the following scores: 1 = coma; 2 = does not turn upright when positioned on the back; 3 = turns upright within 30 s; 4 = minimal ambulatory activity, turns upright within <5 s; and 5 = normal [23].

### Experimental design

Rats (*n* = 66) were included in this study and randomized into sham and pneumococcal meningitis (PM) groups. The rats in the sham group (*n* = 10) were inoculated intracisternally with normal saline, whereas the rats in the PM groups (*n* = 56) were inoculated intracisternally with *S. pneumoniae* and further randomized into four groups (*n* = 14 each) according to the administration of different pretreatments. Prior to induction of meningitis as described above, the rats received an injection once daily for four consecutive days of 7.5 μL recombinant BDNF (6 μg/day and diluted in phosphate-buffered saline (PBS; pH 7.4); PeproTech, Rocky Hill, NJ, USA) [9] or the TrkB inhibitor k252a (1 μg/day and diluted in 10% dimethyl sulfoxide (DMSO) in PBS; Sigma-Aldrich, St. Louis, MO, USA) [24] through a cannula previously implanted in the cerebral ventricle. The equivalent volume of PBS or 10% DMSO was injected into the vehicle control groups. All surviving animals were sacrificed by decapitation at 24 h post-inoculation to harvest the brains. For histological analysis, the animals were perfused through the heart with 50 mL of normal saline, followed by 200 mL of 4% paraformaldehyde (PFA) in PBS, and the brains were removed and post-fixed in 4% PFA overnight at 4 °C. For biochemical analysis, the brains were dissected immediately, and the cortex and hippocampus were isolated and stored at −80 °C.

### Tissue pathology and terminal deoxynucleotidyl transferase dUTP-nick-end labeling (TUNEL) staining

Brain tissues were processed, embedded in paraffin, and cut into coronal sections of 4-μm thickness. Hematoxylin and eosin (H&E; Beyotime, Beijing, China) staining was performed according to a standard protocol, and apoptosis in the hippocampus was examined by TUNEL immunofluorescence staining using an in situ cell death detection kit (Roche, Basel, Switzerland) according to manufacturer instructions.

### Enzyme-linked immunosorbent assay (ELISA)

Brain tissues (hippocampus and cerebral cortex) were homogenized in PBS buffer containing a proteinase inhibitor (100 mg of tissue per milliliter) and centrifuged at 12,000*g* for 15 min. The concentrations of tumor necrosis factor-α (TNF-α), interleukin (IL)-1β, IL-6, and IL-10 in the supernatant were examined using commercially available ELISA kits (eBioscience, San Diego, CA, USA) according to manufacturer protocols. Cytokine and chemokine concentrations in homogenates were normalized to total brain weight and reported as pg/100 mg of tissue.

### Electrophoretic mobility shift assay (EMSA)

Nuclear extract preparation and EMSA were performed using an EMSA kit (Thermo Fisher Scientific, Waltham, MA, USA) to determine NF-κB DNA-binding activity. Briefly, an NF-κB oligonucleotide probe (forward: 5′-AGT TGA GGG GAC TTT CCC AGG C-3′; reverse: 5′-G CCT GGG AAA GTC CCC TCA ACT-3′) was end-labeled with p65 biotin, and 20 μg of nuclear extract was incubated with binding buffer and nonspecific oligonucleotides at room temperature for 15 min, followed by incubation with a p65 biotin-labeled oligonucleotide complex for an additional 15 min. Samples were subsequently separated by electrophoresis in 5.5% polyacrylamide gels in 0.25× Tris-borate-EDTA buffer. Following separation, the bands were detected by chemiluminescence.

### RNA extraction and real-time polymerase chain reaction (PCR)

Total RNA from brain tissues (hippocampus and cerebral cortex) was extracted using Trizol reagent (TaKaRa, Shiga, Japan) and isolated using chloroform according to manufacturer instructions. RNA was converted to complementary DNA (cDNA) using the PrimeScript One Step RT-PCR kit (TaKaRa). Real-time PCR was performed on an ABI7500 system (Applied Biosystems, Carlsbad, CA, USA) using the SYBR Premix Dimmer Eraser kit (TaKaRa) to amplify the cDNA template. The primer sequences were as follows: β-actin forward 5′-GACAGGATGCAGAAGGAGATTACT-3′ and reverse 5′-TGATCCACATCTGCTGGAAGGT-3′; MyD88 forward 5′-GATCCCACTCGCAGTTTGTT-3′ and reverse 5′-GATGCGGTCCTTCAGTTCAT-3′; and TrkB forward 5′-GCTTCTGGAGGGCTTCTCTT-3′ and reverse 5′-TGTTCTCTGGGTCAATGCTG-3′. The primers were synthesized by Shanghai Sangon Biological Engineering Technology Company (Shanghai, China). MyD88 and TrkB gene expression in each sample was normalized to β-actin expression, and the relative expression of messenger RNA (mRNA) was calculated using the 2^−ΔΔCt^ method.

### Western blot

Brain tissues (hippocampus and cortex) were lysed with radioimmunoprecipitation assay buffer containing 1 mM phenylmethylsulfonyl fluoride. The lysates were centrifuged at 12,000×*g* for 15 min at 4 °C, and the protein concentration of each sample was determined using a BCA protein assay kit (Beyotime) according to manufacturer instructions. Protein samples (50 μg) were boiled for 10 min, electrophoresed on 10% sodium dodecyl sulfate polyacrylamide gels, and transferred to polyvinylidene fluoride membranes. The membranes were blocked with 5% skimmed milk in Tris-buffered saline and Tween-20 (TBST) for 2 h at room temperature. The membranes were then incubated at 4 °C overnight with the following primary antibodies: anti-phospho-PI3K, anti-PI3K, anti-phospho-AKT, anti-AKT, and anti-MyD88 (1:1000; Cell Signaling Technology, Danvers, MA, USA), and anti-TrkB (1:1000; Abcam, Cambridge, UK). The membranes were washed with TBST three times for 10 min each and incubated with the appropriate secondary antibody for 1 h. Proteins were visualized by chemiluminescence (Millipore, Billerica, MA, USA) and quantitated using ImageJ software (National Institutes of Health, Bethesda, MD, USA).

### Statistical analysis

Statistical analysis was performed using one-way analysis of variance for parametric data, followed by Tukey’s post hoc test; otherwise, the Mann-Whitney test was used. Survival rates were compared using the log-rank test. If not stated otherwise, values were expressed as the mean ± standard deviation. Differences were considered significant at *p* < 0.05. All graphs were constructed using GraphPad Prism 5.0 software (GraphPad Software, La Jolla, CA, USA).

## Results

### BDNF exerts biological effects following intracerebroventricular delivery

**Fig. 1:**
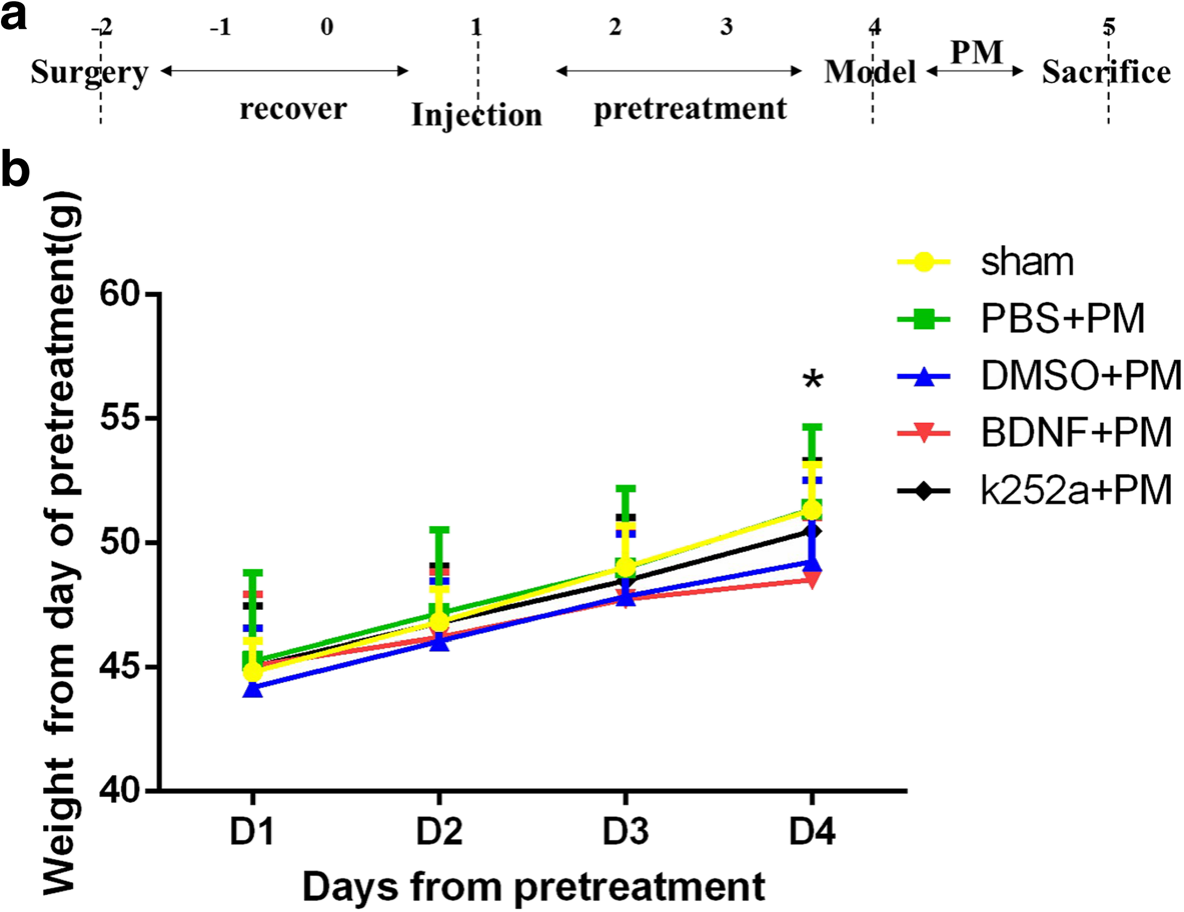
Exogenous BDNF exerts biological effects following intracerebroventricular delivery. **a** Experimental procedures. **b** Rats were weighed from the first day of pretreatment. The body weight of BDNF-pretreated rats increased slowly through day 4, at which time their weight was significantly lower as compared with that of rats in the PBS + PM group. *p < 0.05, BDNF + PM group compared with the PBS + PM group

To assess the effects of BDNF signaling on neuronal protection following infection with PM, the rats were pretreated with exogenous BDNF or the TrkB inhibitor k252a once daily for four consecutive days prior to *S. pneumoniae* exposure. At 24 h post-infection, the rats were sacrificed (Fig. 1a). Previous studies reported that BDNF exerted an effect on CNS neurons associated with regulating food intake [25]; therefore, we used body weight as an indicator to determine the effects of exogenous BDNF delivery by intracerebroventricular injection. The rats were weighed from the first day of pretreatment, and we observed that their body weight following pretreatment with BDNF increased slowly. Moreover, on the final day of pretreatment, their body weight was significantly lower than that of rats in the PBS-treated group (Fig. 1b). These results indicated that exogenous BDNF exerted a biological effect following intracerebroventricular delivery.

### BDNF upregulates TrkB expression

**Fig. 2:**
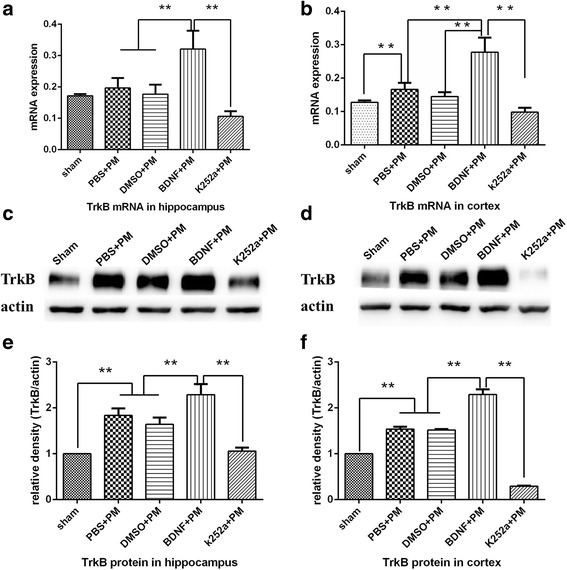
Exogenous BDNF administration upregulated TrkB expression in both the hippocampus and cortex. Real-time PCR analysis of TrkB mRNA levels in the **a** hippocampus and **b** cortex at 24 h post-infection. TrkB mRNA levels significantly increased following infection, with clearly elevated levels observed in the BDNF + PM group. Pretreatment with k252a significantly inhibited TrkB expression relative to levels observed in the BDNF + PM group. Western blot analysis assessed TrkB protein levels in the **c**, **e** hippocampus and **d**, **f** cortex showed significantly increased TrkB protein levels following infection. Administration of exogenous BDNF further increased TrkB expression, which was attenuated by treatment with k252a. **p < 0.01

We used real-time PCR to evaluate TrkB mRNA levels in the hippocampus (Fig. 2a) and cortex (Fig. 2b) at 24 h post-inoculation. Our findings showed that TrkB expression increased following infection and that administration of exogenous BDNF further elevated TrkB expression, which agreed with our previous findings related to TrkB response to exogenous BDNF in a PM model [22]. Pretreatment with the TrkB inhibitor k252a attenuated upregulated TrkB expression as compared with that observed in the BDNF + PM group. Western blot analysis of total TrkB protein levels in both the hippocampus (Fig. 2c, e) and cortex (Fig. 2d, f) showed similar trends as those observed in real-time PCR results. These data confirmed results reported in our previous study showing that exogenous BDNF exerted a neuroprotective role by interacting with its receptor, TrkB, following *S. pneumoniae* meningitis infection [15].

### BDNF reduces the clinical severity of PM

**Fig. 3:**
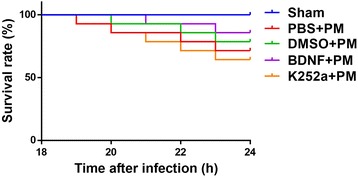
Kaplan-Meier curves showing the survival rates of animals from different groups

As shown in Table 1, all infected animals yielded positive bacterial cultures from CSF samples, whereas no pneumococci grew from CSF samples obtained from the sham group. Moreover, animals pretreated with BDNF showed lower bacterial titers as compared with those from the PBS + PM group, and all infected animals lost weight 24 h after infection as compared with the sham group. Additionally, animals pretreated with BDNF exhibited less weight change relative to that observed in the PBS + PM group, whereas animals pretreated with k252a lost more weight as compared with the BDNF + PM group (Table 1). Clinical score evaluation represents an indicator of the degree of disease severity. Table 1 shows that clinical scores were significantly decreased in all PM groups at 24 h post-inoculation as compared with those measured for the sham group. Additionally, BDNF pretreatment significantly increased the clinical scores of infected rats as compared with those measured in PBS + PM rats. In contrast, rats pretreated with k252a exhibited lower clinical scores as compared with those measured in BDNF + PM rats. These results indicated that BDNF administration attenuated the clinical severity associated with PM. Of the 14 infected animals in each PM group, four (4/14) and three (3/14) animals died in the PBS + PM group and DMSO + PM group, respectively. The survival rate of rats in the BDNF + PM group tended to be higher than that of rats in the PBS + PM group; however, there was no significant difference (*p* > 0.05, log-rank test) (Fig. 3).

**Table 1:** Bacterial titer, change of weight, neurological scores, and mortality in different groups (mean ± standard deviation)

Groups	Bacterial titer, 24 hpi [log_10_ CFU mL^−1^]	Change of weight	Neurological scores^a^	Mortality
Sham	–	−2.64 ± 0.46	5 [5, 5]	0/10
PBS + PM	7.97 ± 0.50	1.50 ± 0.39^*^	3 [3, 4]^*^	4/14
DMSO + PM	7.87 ± 0.42	1.37 ± 0.45^*^	3 [3, 4]^*^	3/14
BDNF + PM	7.35 ± 0.41^**^	1.08 ± 0.36^*,**^	4 [3, 4]^*, **^	2/14
k252a + PM	8.08 ± 0.27^***^	2.28 ± 1.40^*, ***^	3 [3, 4]^*, ***^	5/14

Bacterial titer, change of weight, neurological scores, and mortality were evaluated at 24 h post-infection

*p < 0.05, compared to sham group

**p < 0.05, compared to PBS + PM group

***p < 0.05, compared to BDNF + PM group

^a^Median [minimum, maximum]

### BDNF alleviates the pathologic severity of PM

**Fig. 4:**
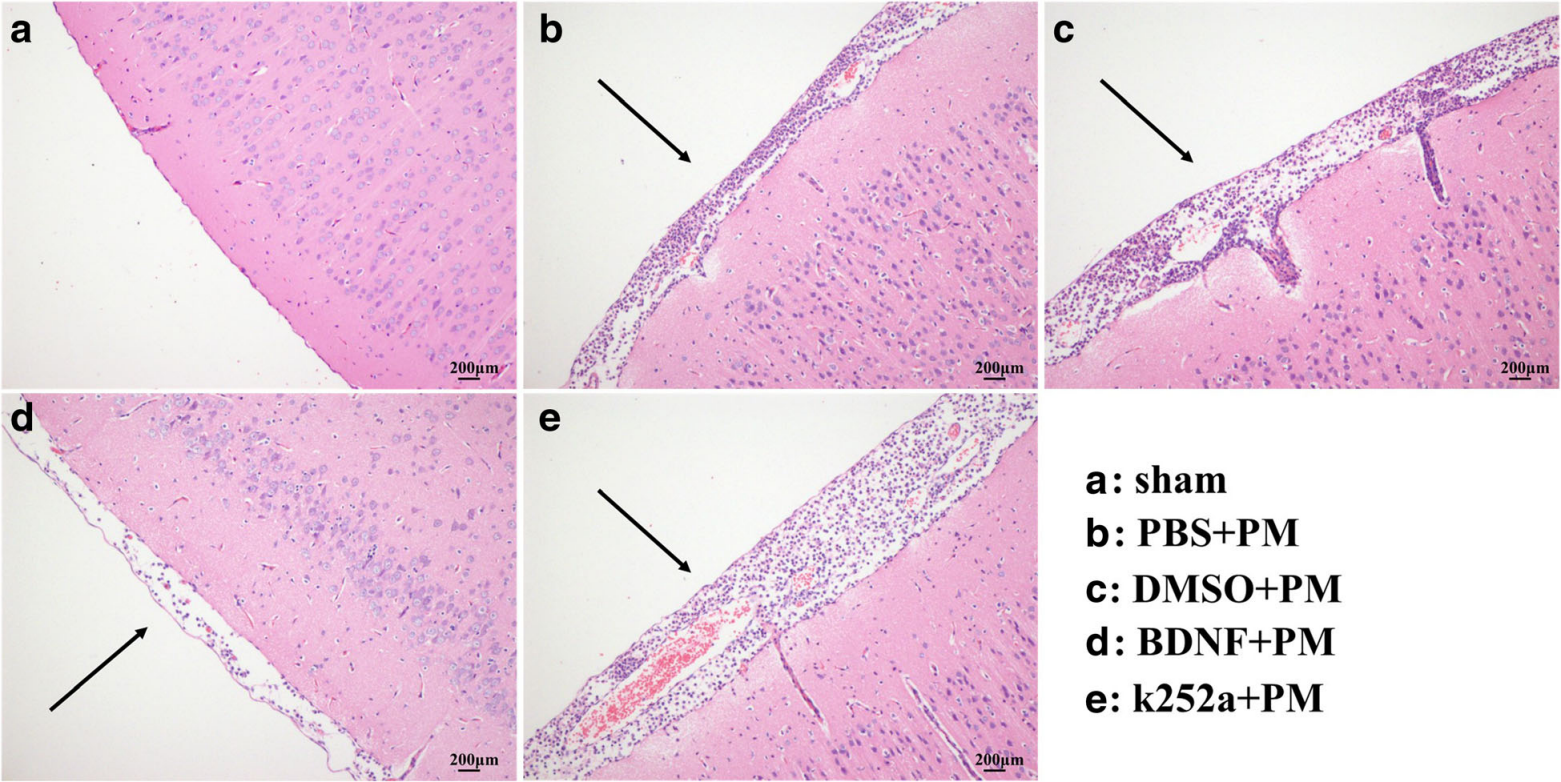
BDNF alleviates the pathological severity of PM. **a** Mice in the sham group showed no inflammatory infiltrates, whereas **b**–**e** H&E-stained sections showed infiltration of inflammatory exudate and mononuclear cells in the subarachnoid space of infected mice. The BDNF + PM group showed a mild inflammatory response as compared with that observed in the PBS + PM group

We investigated morphological changes according to H&E staining at 24 h post-infection. As shown in Fig. 4, large amounts of inflammatory exudate and infiltrated cells accumulated in the subarachnoid space of all infected groups. Additionally, a significant decrease in the number of cells infiltrated with inflammatory factors was observed in the BDNF + PM group as compared with that observed in the PBS + PM group, although there was no obvious difference between the k252a + PM group and the DMSO + PM group. These results illustrated the protective effect of BDNF signaling on the pathologic severity of PM.

### BDNF administration downregulates proinflammatory cytokine expression and upregulates anti-inflammatory cytokine expression associated with PM infection

**Fig. 5:**
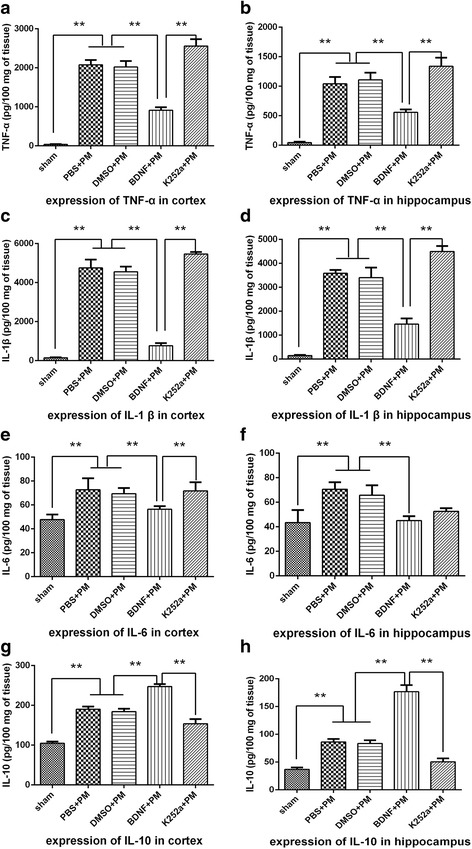
BDNF downregulates proinflammatory cytokine expression and upregulates anti-inflammatory cytokine expression in PM. Levels of proinflammatory cytokines (TNF-α, IL-1β, and IL-6) and anti-inflammatory cytokines (IL-10) in both the hippocampus and cortex at 24 h post-infection as measured by ELISA. Levels of both proinflammatory and anti-inflammatory cytokines were significantly increased at 24 h post-infection as compared with levels observed in the sham group. Administration of exogenous BDNF significantly decreased the levels of TNF-α, IL-1β, and IL-6 in the **a**, **c**, **e** hippocampus and **b**, **d**, **f** cortex, and **g**, **h** further elevated IL-10 levels. Rats pretreated with k252a exhibited enhanced expression of proinflammatory cytokines and attenuated expression of anti-inflammatory cytokines as compared with levels observed in the BDNF + PM group. **p < 0.01

Brain homogenates were examined for cytokine (TNF-α, IL-1β, IL-6, and IL-10) levels by ELISA. The levels of all inflammatory cytokines analyzed were significantly elevated in all the infected rats as compared with those in the sham group. However, the expression of proinflammatory cytokines (TNF-α, IL-1β, and IL-6) in the hippocampus (Fig. 5a, c, and e) and the cortex (Fig. 5b, d, f) was significantly reduced in the BDNF + PM group as compared with that observed in the PBS + PM group, whereas rats pretreated with k252a showed elevated inflammatory factor expression relative to that observed in the BDNF + PM group (except for IL-6 in the hippocampus; Fig 5e). In contrast, expression of the anti-inflammatory cytokine IL-10 was significantly elevated in both the hippocampus (Fig. 5g) and cortex (Fig. 5h) in the BDNF + PM group as compared with that observed in the PBS + PM group, whereas k252a pretreatment exerted the opposite effect as that observed following BDNF pretreatment. These findings indicated that BDNF/TrkB signaling was significantly associated with the expression of cytokines/chemokines in response to PM infection.

### BDNF inhibits the MyD88/NF-κB-signaling pathway associated with PM

**Fig. 6:**
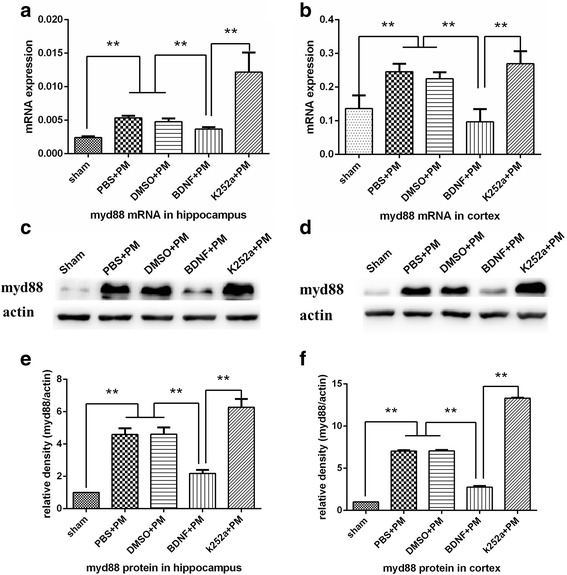
BDNF inhibited MyD88 expression in PM. MyD88 mRNA levels in the **a** hippocampus and **b** cortex increased at 24 h post-infection. BDNF pretreatment inhibited the upregulation of MyD88 mRNA, whereas rats pretreated with k252a showed increased MyD88 expression as compared with that observed in the BDNF + PM group. Western blot analysis confirmed lower levels of MyD88 protein in the **c**, **e** hippocampus and **d**, **f** cortex following BDNF treatment, whereas these results were not observed in the k252a + PM group. **p < 0.01

The MyD88/NF-κB-signaling pathway plays an important role in *S. pneumoniae* meningitis infection. MyD88 is a critical molecule associated with initiation of host immune response associated with PM [26]. Here, we used real-time PCR to evaluate MyD88 mRNA levels in the hippocampus and cortex at 24 h post-inoculation and found significant increases in MyD88 mRNA levels in both the hippocampus (Fig. 6a) and cortex (Fig. 6b) of all infected rats. Interestingly, BDNF pretreatment significantly prevented PM-related increases in MyD88 mRNA levels in brain tissues, whereas pretreatment with k252a significantly increased MyD88 expression as compared with levels observed in the BDNF + PM group. Western blot analysis to evaluate total MyD88 protein levels in the hippocampus (Fig. 6c, e) and cortex (Fig. 6d, f) agreed with mRNA levels measured by real-time PCR.

**Fig. 7:**
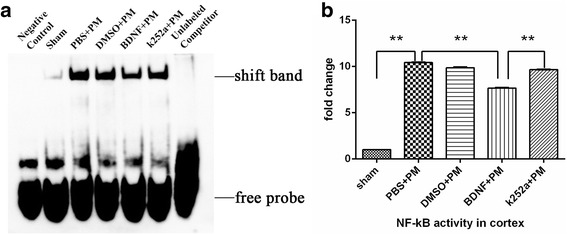
BDNF reduces NF-κB-binding activity in the cortex at 24 h post-infection. **a** NF-κB DNA-binding activity examined by EMSA revealed increased NF-κB-binding activity in all infected mice relative to levels observed in the sham group. BDNF pretreatment inhibited NF-κB-binding activity as compared with levels observed in the PBS + PM group, and pretreatment with k252a significantly increased NF-κB-binding activity as compared with activity observed in the BDNF + PM group. **b** Quantitative analysis of the EMSA results. **p < 0.01

NF-κB is an important transcription factor that plays a key role in the pathophysiological mechanism associated with PM. We performed an EMSA to determine whether exogenous BDNF effects NF-κB-related DNA-binding activity. As shown in Fig. 7a, NF-κB-binding activity in the cortex increased at 24 h post-inoculation in all PM groups as compared with that in the sham group. Interestingly, BDNF pretreatment significantly prevented PM-associated elevations in NF-κB-related DNA-binding activity as compared with the PBS + PM group, and pretreatment with k252a significantly increased NF-κB-binding activity as compared with activity observed in the BDNF + PM group, although there was no statistical difference between the binding activity observed between the DMSO + PM group and the k252a + PM group (Fig. 7b). These results indicated that BDNF/TrkB signaling inhibited activity associated with the MyD88/NF-κB-signaling pathway associated with PM.

### BDNF reduces hippocampal apoptosis in PM

**Fig. 8:**
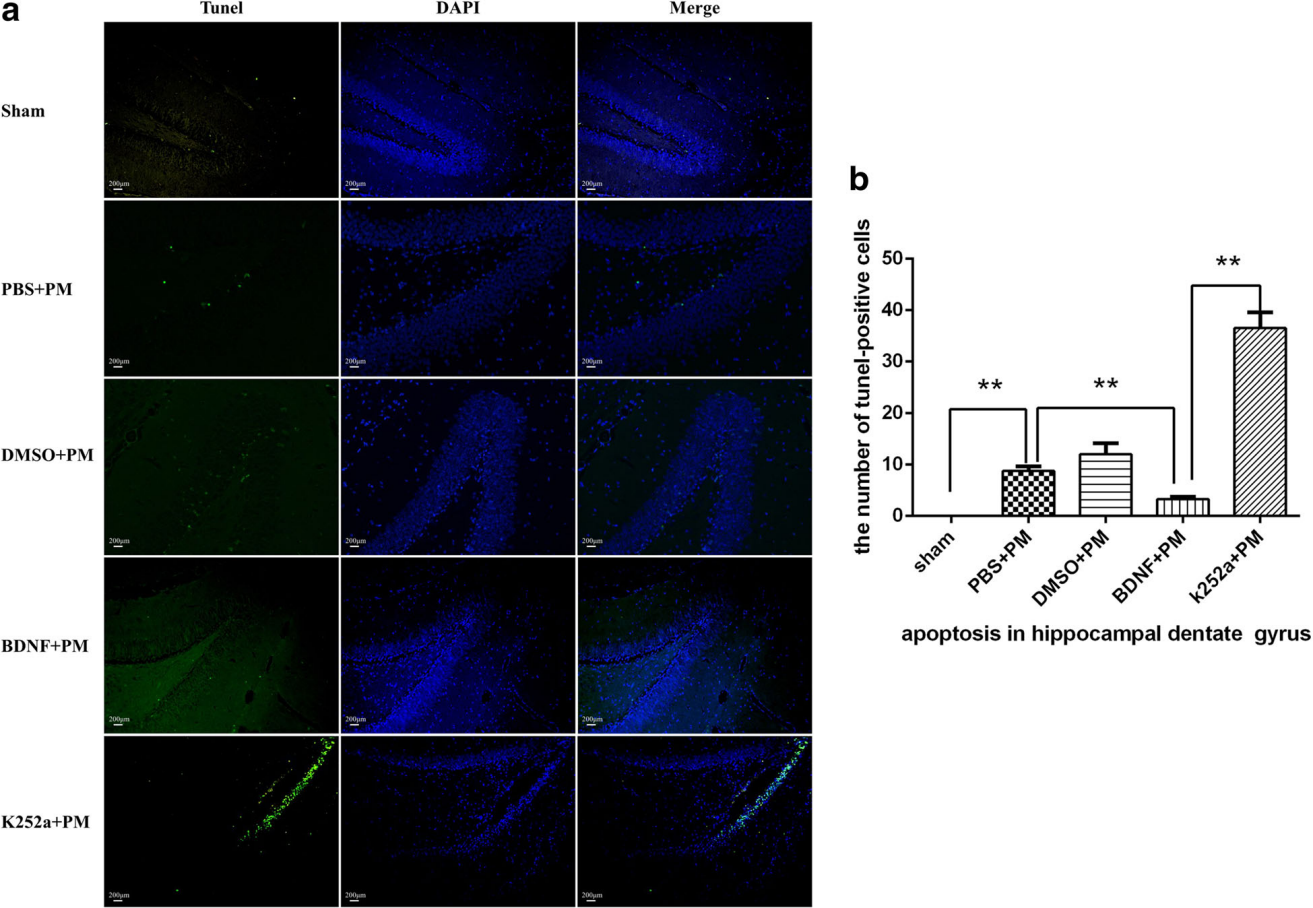
BDNF reduces hippocampal apoptosis in PM. Apoptotic neurons in the hippocampal dentate gyrus were assessed by TUNEL staining. **a** BDNF administration prevented neurons from apoptotic injury from PM-induced hippocampal apoptosis, whereas k252a-pretreatment significantly elevated levels of hippocampal apoptosis as compared with levels observed in the BDNF + PM group. **b** Quantitative analysis of the TUNEL results. **p < 0.01

Hippocampal apoptosis was investigated by TUNEL staining. Our results indicated that all PM groups exhibited increased levels of apoptosis as compared with those in the sham group, with most apoptotic cells mainly located in the inner layer of the dentate (Fig. 8a). Furthermore, rats that received BDNF pretreatment showed lower levels of apoptosis in the hippocampal dentate gyrus as compared with the PBS + PM group, and rats pretreated with k252a showed an obviously increased number of apoptotic cells as compared with that observed in the BDNF + PM group (Fig. 8b).

### BDNF activates the PI3K/AKT-signaling pathway in PM

**Fig. 9:**
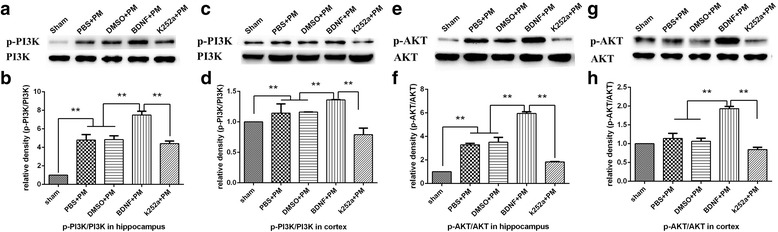
BDNF activates the PI3K/AKT-signaling pathway in brain tissue 24 h post-infection with PM. Levels of phosphorylated PI3K and AKT in the **a**, **b**, **e**, **f** hippocampus and **c**, **d**, **g**, **h** cortex as examined by western blot using specific antibodies. BDNF administration increased levels of phosphorylated PI3K and AKT, whereas k252a pretreatment decreased these levels. **p < 0.01

To examine whether exogenous BDNF activates PI3K/AKT signaling in PM, we measured the levels of phosphorylated PI3K (p-PI3K) and AKT (p-AKT). Figure 9 shows that levels of p-PI3K and p-AKT were significantly increased in infected brain tissues as compared with those observed in the sham control, although the total protein levels of PI3K and AKT were unchanged. Additionally, administration of exogenous BDNF significantly increased p-PI3K and p-AKT levels in both the hippocampus (Fig. 9a, b, e, f) and cortex (Fig. 9c, d, g, h) as compared with the levels observed in the PBS + PM group. However, rats pretreated with k252a exhibited significantly lower levels of PI3K and AKT phosphorylation relative to the levels observed in the BDNF + PM group. These data indicated that BDNF/TrkB signaling activated the PI3K/AKT-signaling pathway in PM via PI3K and AKT phosphorylation.

## Discussion

In this study, we observed that BDNF pretreatment reduced the clinical and pathological severity of PM and also alleviated the inflammatory response and hippocampal apoptosis associated with *S. pneumoniae* meningitis. Additionally, we demonstrated that downregulated BDNF/TrkB interactions following treatment with a TrkB-specific pharmacological inhibitor reversed the effects observed following BDNF treatment. Furthermore, based on investigation of intracellular signaling pathways, our results indicated that the neuroprotective effects exerted by BDNF might occur through modulation of the MyD88/NF-κB- and PI3K/AKT-signaling pathways.

Despite the advances in antimicrobial agents and improved neurological management, it remains difficult to improve outcomes for children suffering from bacterial meningitis [27]. The discovery of the neuroprotective effects associated with BDNF in diverse CNS-related diseases offers a potentially promising strategy for treating paradigms of acute neuroinfection. Although BDNF expression is increased transiently in the brains during the acute phase of meningitis [15], this endogenous protective mechanism is insufficient to protect neurons from infection-related death. Early studies reported that adjuvant therapy involving BDNF protects the brain from three distinct forms of injury in experimental bacterial meningitis [28]; however, here, we investigated the effects of BDNF/TrkB interaction on the inflammatory response and hippocampal apoptosis, as well as its effect on other potential intracellular signaling pathways responsible for this protective activity associated with PM.

During the acute phase of the disease, inflammatory mediators are produced and released by brain-resident immune cells and different neuron populations, thereby contributing to disease pathogenesis [29, 30]. Proinflammatory cytokines, such as TNF-α, IL-1β, and interferon-γ, play a key role in disease progression during diverse types of CNS insult, eventually resulting in depleted energy production, cell death, and brain injury [31–33]. In this study, we observed that that BDNF treatment decreased both mRNA and protein levels of TNF-α, IL-1β, and IL-6 induced by the presence and recognition of bacterial components. These results might partially explain the reduced pathological severity observed in our H&E staining results. IL-10 is an anti-inflammatory cytokine that exhibits neuroprotective effects based on studies reporting that exogenous administration of IL-10 protects the brains from ischemic injury [34] and increased severity of brain injury in IL-10 knockout mice suffering from multiple sclerosis [35]. Here, exogenous BDNF administration increased IL-10 expression at the transcription and translation levels, suppressed proinflammatory cytokine expression, and enhanced anti-inflammatory cytokine expression in bacterial meningitis models, which was consistent with observations from previous studies [19, 36]. A recent in vivo study showed that BDNF was capable of regulating intracellular signaling molecules to inhibit inflammatory cytokine expression induced by peptidoglycan in human dental pulp cells [37]. Despite these findings, the exact mechanism by which BDNF regulates anti-inflammatory effects during bacterial meningitis infection remains unclear.

The MyD88/NF-κB complex forms a classic inflammatory signaling pathway critical to responses to CNS insult. BDNF prevents neurological deficiency in focal cerebral ischemia by suppressing the toll-like receptor/MyD88-signaling pathway [14], and in PM, the MyD88/NF-κB-signaling pathway is activated by recognition of bacterial compounds [38]. Here, we observed that BDNF pre-treatment inhibited the expression of MyD88 and nuclear translocation of NF-κB, thereby neutralizing the detrimental effects of excessive inflammatory responses in suggesting that BDNF administration might enable preservation of neurons from PM-related injury.

Previous studies revealed three major forms of brain injury according to PM-related histopathology results: necrosis of the cortex, apoptosis in the hippocampus, and neuronal loss in the spiral ganglion [28]. The hippocampal dentate gyrus is particularly vulnerable to apoptotic injury during PM and usually appears ~18 to ~24 h post-infection and is accompanied by massive increases in the number of apoptotic cells [39], with most apoptotic cells located in the inner layer of the dentate gyrus, which is consistent with our results [40]. The neuroprotective effects of BDNF were described in a study focused on neonatal hypoxia-ischemia, wherein BDNF prevented hippocampal apoptosis by blocking caspase-3 activation [41]. BDNF/TrkB interaction induces complex intracellular signal-transduction cascades, including the mitogen-activated protein kinase pathway, PI3K/AKT pathway, and phospholipase C-gamma pathway [42]. Additionally, previous studies reported that BDNF delivered by bone marrow stem cells inhibited apoptosis in both multiple sclerosis and ischemic stroke models through upregulated expression of anti-apoptotic B cell lymphoma 2 (BCL-2) and downregulated expression of pro-apoptotic BCL-2-associated X protein (BAX). Because PI3K/AKT signaling occurs upstream of BCL-2/BAX expression/repression, we hypothesized that the anti-apoptotic effects associated with BDNF/TrkB interactions might involve the PI3K/AKT pathway. The results of our study showed that PI3K/AKT signaling was significantly upregulated after meningitis infection and that this activity was enhanced following BDNF pretreatment. As an additional pathway associated with cell survival, excess concentrations of cellular calcium represent a common pathophysiological phenomenon during bacterial meningitis [43]. Activity associated with PI3K/AKT signaling is capable of regulating calcium channels to enhance their capacity to bind intracellular calcium, thereby promoting cellular calcium homeostasis and survival [44].

## Conclusions

In summary, our findings indicated that BDNF/TrkB interactions resulted in anti-inflammatory and anti-apoptotic effects in experimental models of PM likely through regulation of the MyD88/NF-κB- and PI3K/AKT-signaling pathways. These results suggested that BDNF might constitute a promising therapeutic alternative for the attenuation of PM-associated neurofunctional deficits.

## Abbreviations

AKT: Protein kinase B
BDNF: Brain-derived neurotrophic factor
CNS: Central nervous system
CSF: Cerebrospinal fluid
DMSO: Dimethyl sulfoxide
ELISA: Enzyme-linked immunosorbent assay
EMSA: Electrophoretic mobility shift assay
ERK: Extracellular signal-related kinase
H&E: Hematoxylin and eosin
IL: Interleukin
MyD88: Myeloid differentiation primary response gene 88
NF-κB: Nuclear factor kappa B
PBS: Phosphate-buffered saline
PCR: Polymerase chain reaction
PFA: Paraformaldehyde
PI3K: Phosphatidylinositol 3-kinase
PM: Pneumococcal meningitis
*S. pneumoniae*: *Streptococcus pneumoniae*
TBST: Tris-buffered saline and Tween-20
TNF-α: Tumor necrosis factor-α
TrkB: Tropomyosin-receptor kinase B
TUNEL: tissue pathology and terminal deoxynucleotidyl transferase dUTP-nick-end labeling
